# Bioaccessibility by perspiration uptake of minerals from two different sulfurous peloids

**DOI:** 10.1007/s10653-023-01639-z

**Published:** 2023-06-21

**Authors:** Carla Marina Bastos, Fernando Rocha, Carla Patinha, Paula Marinho-Reis

**Affiliations:** 1 Department of Geosciences, GeoBioTec Research Centre, University of Aveiro, 3810-193 Aveiro, Portugal; 2 Institute of Earth Sciences (ICT) – Pole of the University of Minho, University of Minho, 4710-057 Braga, Portugal; 3 Exatronic, Lda, Aveiro, Portugal

**Keywords:** Clays, Mineral-medicinal water, Peloids, Artificial perspiration, Transdermal delivery

## Abstract

The risks associated with the use of peloids in thermal centers, spas, or at home, must be tested to develop appropriate safety guidelines for peloids formulations and the release of substances of high concern. Additionally, the beneficial effects of some elements on human health should be assessed to aid in interpreting the therapeutic action and effectiveness of pelotherapy on dermatological or osteomuscular disorders. Therefore, a methodology was developed to better understand the biogeochemical behavior of the elements in formulated peloids. Two peloids were formulated with the same clay and two different sulfurous mineral-medicinal waters for 90 days, with light stirring every 15 days. Bentonite clay, with a high content of smectite and Ca and Mg as the main exchangeable cations, and high heat capacity, was used. The selected mineral-medicinal waters were collected from two Portuguese thermal centers with recognized therapeutic efficacy for rheumatic, respiratory and dermatological pathologies. The peloids were used without drying and withdrawn directly from the maturation tank, and a mixture of bentonite and demineralized water was prepared as a reference sample. A stabilized, ready-to-use, artificial perspiration test was used to simulate the peloids’ interaction with skin. Thirty-one elements extracted from the two prepared peloids were analyzed using ICP-MS. The data were analyzed and related to the mineralogical composition of the original clay and supernatant composition of the maturation tanks. The content of some potentially toxic elements and metals’ bioaccessibility by perspiration showed very low solubility and undetectable amounts extracted from the studied samples. This analytical method provided reliable information on dermal exposure and the identification of some elements that may enter the systemic circulation, requiring implementation of surveillance and control measures.

**Graphical abstract:**

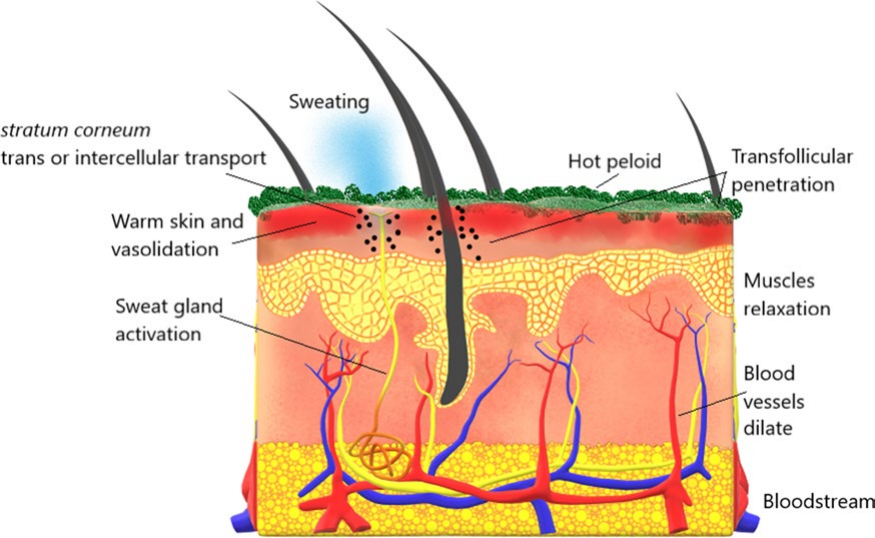

**Supplementary Information:**

The online version contains supplementary material available at 10.1007/s10653-023-01639-z.

## Introduction

Pelotherapy is a therapeutic and wellness modality that is commonly used in thermal centers, spas, or even in natural settings. Muds, also known as peloids, is frequently used for its therapeutic effects in treating dermatological and osteomuscular pathologies, despite the lack of knowledge of their exact effects on health, as exhibited in several studies (Espejo et al., 2012; Fioravanti et al., 2017; Forestier et al., 2016; Ortega et al., 2017).

According to recent definitions, peloids are a mixture of geomaterials with mineral-medicinal water or salt water, sometimes enhanced with biological compounds like algae, diatoms, botanical species or with artificial components for fragrance, color, or other technological benefits resulting from peloid artificial manipulation. Peloids are applied to the skin through bathing or localized application in the form of packs or compresses [Gomes, 2018 and references therein]. Natural peloids are typically applied in thermal and wellness settings, where the skin contact procedure is based on the definition of peloid temperature and treatment time. Most of these treatments occur at 40 °C and 45 °C, during a period of 20 to 30 min, and may be complemented with other thermal and spa modalities (e.g., Crenobalneotherapy). However, some of these muds are also available on the market as natural products, despite being artificially improved and sold as self-care dermocosmetics (Moraes et al., 2017).

Peloids are topically applied substances, and several authors have suggested that temperature and biological composition play a crucial role in the penetration pathway through the skin barrier (Cervini-Silva et al., 2015; Quintela et al., 2013; Poli et al., 2009). The sudation effect and simultaneous vasodilation of the skin are the explanations for this phenomenon (Quintela et al., 2012). However, there is no evidence to support the notion that clay chemical elements can pass through an intact skin barrier or reach cells through transfollicular penetration.

Peloids are used as face masks or mud baths, and there is no established ideal amount of product to be used per application. The application indications for commercial peloids available on the market vary, and thermal centers have their own therapeutic protocols, some of which with historical references and are still used in a traditional way (Bastos & Rocha, 2022).

Considering the European Regulation for cosmetics, there are no specific recommendations for natural peloids. Most of the natural peloids developed in thermal centers continue to use old formulations, neglecting the importance of human safety compliance during use. Despite the lack of detailed knowledge regarding the hazardous and beneficial effects on human health, pelotherapy remains advantageous. Exposure to clay chemical elements through transdermal transmission causes fewer side-effects compared to the mobility of these elements after clay ingestion (Tateo & Summa, 2007).

There are a few controlled studies focused on the toxicology of natural peloids and the interface behavior between the skin and the chemical–mineralogical constituents of peloids (Gerencsér et al., 2010; Tateo et al., 2009). The effect of beneficial elements through transdermal delivery is also relevant (Tateo & Summa, 2007). Rossi et al*.* (2013) evaluated the quality of Euganean Thermal mud (Italy), correlating the degree of maturation and its affinity with the skin. They used a non-invasive method based on the permutability principle of tensiometry, demonstrating correlations between peloid´s chemical–mineralogical characteristics and its surface. Gerencsér et al*.* (2010) focused their work on natural Hungarian peloids and studied the possible biological toxicity of two peloids used in medicinal treatments. They used organisms to carry out their ecotoxicological tests, the earthworm Eisenia and the White mustard seedling growth test. The endpoints for Eisenia were lethality, mass growth and reproductive capacity, and for white mustard, the end point was length. The results showed different effects of the peloids on earthworms and mustard seeds, and the authors considered the presence of soluble substances in those peloids as the main reason for such difference. Tateo et al. (2009) used experiments to evaluate the skin’s permeation to the transdermal delivery of essential elements from peloids. The methodology used for transdermal evaluation was a sweat leaching method and in vitro permeation experiment, using the Franz diffusion cells method. The results obtained showed that after 20 min of mud application several chemical elements were available to cross the skin. However, the authors questioned the caution criteria for transdermal hazardous element concentrations establishment, the effectiveness of transferring the chemical elements by the skin for people under cure, and the need for clinical observation of the peloids' biological effect.

Carretero et al. (2010) used an artificial sweat solution to evaluate the mobility of essential and toxicological elements to the epidermis from four Spanish peloids. They simulated the action of thermal therapy for temperatures of 45 °C (± 2 °C) and identified a peloid with the highest concentration of soluble elements whose potential toxicity was negligible.

The lack of scientific evidence and knowledge about the cytotoxic effects of natural clays used in peloid formulations and the impact of the mineral-medicinal water on the enrichment of the final product combined with the life cycle of the peloid use (some thermal centers recycle the peloids used), creates the urgent need for further studies to support the definition of quality parameters and protocols, and guarantee the balance between risk and benefit.

The toxicological profile of potentially toxic elements in peloids and their adversity, and essential elements and beneficial health effects, should be understood by their toxicokinetic behavior when in contact with the skin. The significance of the exposure assessment, regarding “normal or reasonably foreseeable conditions of use” (as defined in Article 3 EC No. 1223/2009), depends on the users’ perspective and the chemical specifications of the elements (EC Regulation, 2009).

The most characteristic effect of long-term dermal exposure to toxicological elements is the development of skin lesions or allergies (WHO, World Health Organization, 2014), but there are other kinetic interactions, such as absorption, distribution, metabolism or excretion, that can be relevant to evaluate the mechanisms of action and toxicological significance. At the same time, attention should be paid to the essential elements and the health benefits of the presence of some elements in the healing process of some diseases, as we can find in some clinical studies where physical–chemical aspects are enhanced as the main drivers of the results (Cozzi et al., 2018; Gáti et al., 2018; Huber et al., 2019; Karagülle et al., 2018; Ortega et al., 2017).

Metals are present naturally in clays and mineral-medicinal waters, the raw materials necessary for peloid development. The presence of elements in excessive amounts, such as lead, cadmium, arsenic, nickel, copper, iron, chromium, cobalt and aluminum, is a concern for compliance with cosmetic regulations, before being placed on the market and during cosmetovigilance. Several studies show the presence of toxic metals in commercially available cosmetics in amounts that can create danger to human health (Borowska & Brzóska, 2015; Bruzzoniti et al., 2017).

The aim of this study was to provide a safety evaluation of two sulfurous peloids, by using a harmonized test that can provide conclusive results about the migration values of the clay chemical elements and their toxic or wellness potential.

## Materials and methods

### Peloids

Two peloid formulations were designed based on previous research indicating the potential use of a Portuguese bentonite clay (“Benavila”) in pelotherapy (Rebelo et al., 2011), as an active agent (Dziadkowiec et al., 2017) and two different mineral-medicinal waters. The formulations included this clay as an active ingredient and two different types of mineral-medicinal waters collected from the water sources catchment that supply the Cró Hotel & Thermal Spa (CRO) and Caldas da Rainha Thermal Hospital (CR) in Portugal (Rebelo et al., 2015). The peloids were left to mature for 90 days (Table 1), with stirring every 15 days, under natural light and at a room temperature of 22 °C ± 3 °C. The pH measured remained around 6 throughout the maturation period (Aguzzi et al., 2013).

**Table 1:** Samples used in the study

Sample	Maturation time	Solid phase + Liquid phase
CRO1	90 days	Bentonite + Cró mineral water
CR1	90 days	Bentonite + Caldas da Rainha mineral water
BV3	–	Bentonite + demineralized water

The Caldas da Rainha sulfurous water is characterized for its hypersaline mineralization, with chloride and sodium being the predominant ions. It has an intense sulfuric odor and taste, and a total mineralization of about 2742 mg/L, as reported in the LAIST/ISQ Analysis Report No 17138-18, dated July 2nd, 2018. The Cró sulfurous water has a low mineralization level (around 372 mg/L) with bicarbonate and sodium as predominant ions, as stated in the LAIST/ISQ Analysis Report No 30680-17, dated November 7th, 2017. The anion and cation concentrations for both mineral-medicinal waters are presented in Table 2. The Caldas da Rainha mineral-medicinal water has a pH of 6.7, while the pH of Cró mineral-medicinal water is 8.7.

**Table 2:** Thermal water chemical analysis

		CRÓ^a^	CALDAS DA RAINHA^b^
*Anions*			
Bicarbonate	mg(HCO_3_)/L	153	319
Carbonate	mg(CO_3_)/L	< 2	< 2
Chloride	mg/L	31	930
Fluoride	mg/L	15	1.2
Hydrogen sulfide	mg(HS)/L	2.8	3.7
Nitrate	mg(NO_3_)/L	< 0.30	< 0.30
Nitrite	mg(NO_2_)/L	< 0.010	< 0.010
Silicate	mg(H_3_SiO_4_)/L	< 1	< 1
Sulfate	mg(SO_4_)/L	20	530
*Cations*			
Ammonia nitrogen	mg(NH_4_)/L	0.05	0.38
Calcium	mg/L	3.5	261
Lithium	mg/L	0.6	< 0.10
Magnesium	mg/L	0.21	54
Potassium	mg/L	2.5	4.7
Sodium	mg/L	95	621
Iron	mg/L	< 0.010	0.034

^a^LAIST/ISQ Analysis Report No 30680-17, 2017.11.07

^b^LAIST/ISQ Analysis Report No 17138-18, 2018.07.02

The therapeutic indications for Caldas da Rainha and Cró mineral medicinal water are for rheumatic and respiratory pathologies, and Cró is also recommended for dermatological diseases.

The Benavila bentonite (BV3), with a particle size fraction of < 63 µm (determined by wet sieving), was prepared using demineralized water (in a 1:2 ratio) as a reference control. Samples were then dried in an oven at 50 °C before undergoing mineralogical, chemical and morphological characterization. The clay fractions (< 2 µm) were obtained by sedimentation, using Stokes law.

### Artificial perspiration

The mechanisms of artificial perspiration tests have been used to mimic natural human eccrine perspiration. For this study, a stabilized ready-to-use artificial perspiration formulation with a pH of 6.5, from Pickering Laboratories (Lot No 903026), was selected to comply with EN 1811:2011 + A1:2015. This standard specifies a test method for simulating the release of nickel to determine compliance with Annex VII of the REACH directive. The solution contained water (98–99%), sodium chloride (≤ 1.0%) and Urea (≤ 0.5%). EN 1811:2011 provides a simple reporting process of results for metal items that come into direct or prolonged contact with the skin. This harmonized test can provide conclusive results on migration values and clarify the assumptions and constraints related to compliance and non-compliance criteria.

### Mineralogical characterization of the peloids

The mineralogical composition of the samples was determined using X-ray diffraction (XRD) with a Philips/PANalytical X’Pert-Pro MPD diffractometer, with Cu Kα radiation (*λ* = 1.5405 Å), and a step size of 0.02° 2*θ* s^−1^, in the 4–65° 2*θ* range.

To identify clay minerals, on orientated aggregates of the clay fractions (< 2 µm), a suspension was prepared and dropped on glass slides and air-dried. XRD scans were run in these air-dried glass slides, and then, after glycolate saturation (24 h), a final submission to heat (at 500 °C), to better differentiate the clay minerals.

The semi-quantification of clay minerals was performed by measuring the peak areas of the basal reflections on the obtained diffractograms (background correction and peak area measurement done by X’Pert-Pro MPD software) and the peak intensities corrected using specific reflection powers (Galhano et al., 1999; Martins et al., 2007; Oliveira et al., 2002) and supported by chemical analyses data. The total proportion of clay minerals in each sample was first determined by considering the peak area of the diffraction maximum of the phyllosilicates at 4.48 Å in unoriented preparations. The percentages of different clay minerals were then obtained from the peak areas of the diagnostic basal reflections of the species in the oriented aggregates.

### Chemical composition of the supernatant from the maturation tanks

The supernatant was collected after 90 days of maturation, and the concentrations of major and trace elements were determined using an Inductively Coupled Plasma-Mass Spectrometry (ICP-MS) Agilent Technologies 7700 Series equipped with nickel sampler and skimmer cones and a collision/reaction cell. Calibrations for the ICP-MS were prepared using multi-element certified standards and verified using an independent certified standard. A rigorous quality control program was implemented for element determination, including method blanks and replicate samples. Precision was estimated by calculating the relative standard deviation (RSD) of three replicate samples and was found to be ≤ 10%. The detection limits (d.l.) were calculated as three times the standard deviations of the blanks (*n* = 10).

### Morpho-chemical characteristics of the peloids

The chemical composition of the samples was determined using X-ray fluorescence (XRF), on a Philips PANalytical AX-IOS PW 4400/40 fluorescence spectrometer. Loss on ignition (LOI) was also acquired by heating 1 g of sample at 1000 °C for 1 h in a furnace.

The cation exchange capacity (CEC) was estimated by the ammonium acetate method (Quintela et al., 2012; Rebelo et al., 2011) and the exchangeable cations (Na^+^, K^+^, Mg^2+^ and Ca^2+^) were determined using ICP-MS (Agilent Technologies 7700 Series).

Micromorphological analysis was also performed on the clay fraction (< 2 µm) of the Benavila bentonite, CRO and CR peloids using an ultra-high-resolution analytical scanning electron microscope, HR-FESEM Hitachi SU-70, to detect some textural changes in the mineral matrix. Chemical analyses were carried out on the surface of the clay particles using energy-dispersive X-ray spectroscopy (EDS). For these analyses, a clay powder fraction was fixed onto a carbon sticker and covered with graphite film.

### Perspiration testing procedure

Following the proposal of Carretero and co-authors (2010), the samples were used in their natural state in the maturation tank. Five grams of each sample was mixed with 50 mL of perspiration solution and then stirred at 60 rpm for 1 h at 45 °C (± 2 °C). Subsequently, the slurry was centrifuged at 4000 rpm for 20 min to separate the reacted solution, which was kept in glass containers. Prior to testing, the solutions were acidified to 1.5% v/v with concentrated nitric acid. The resulting extract’s chemical composition was determined using ICP-MS and compared to that of the ready-to-use artificial perspiration solution (APS).

The artificial perspiration experiments were performed in triplicate as technical replicates. Statistical data analysis for each sample was displayed as mean ± standard error of the mean (SE) for a population sample size of *n* = 3.

### Nickel and chromium systemic toxicity evaluation

The determination of exposure to nickel (Ni) and chromium (Cr) released from CRO1 and CR1 peloids was based on compliance with current cosmetic legislation, specifically Regulation (EC) No 1223/2009 and the “Notes of Guidance for the Testing of Cosmetic Ingredients and Their Safety Evaluation” by the Scientific Committee on Consumer Safety (SCCS, 2021). These metals are listed in Annex II of the Regulation (EC) No 1223/2009. The SCCS uses relevant data of cosmetic ingredients with potential concern for human health, provided by open literature and other relevant sources, considering universal risk assessment processes and scientifically valid toxicity testing procedures.

The safety evaluation procedure comprised four steps, namely hazard identification (e.g., Ni and Cr), exposure assessment (determination of the amount of substance and frequency of use), dose–response assessment (evaluation of the relationship between the exposure and toxic response) and risk characterization.

Peloids are not covered as product category by SCCS (2021), and an amount per day (*q*_*x*_) is not given. As suggested by the SCCS, for other cosmetic product categories, a case-by-case assessment of the daily exposure level and frequency of application needs to be made. In this study, for a daily exposure, the data proposed as cosmetic products default values for “body packs: mud bath/clay bath” were considered for an exposure scenario as follows: for an application frequency of 4 times/year, 416 g per application use, for 15 670 cm^2^ (body surface area-head surface area), for an exposure time of 20 min and washing the skin with water when the treatment is finished (Höglund et al., 2012).

The dermal exposure models proposed by SCCS (2021) for dermal exposure calculation consider that only a fraction of the product is retained on the skin. A retention factor, **f**_**ret**_, represents the fraction available for uptake. For peloids application it was considered the retention factor for ‘skin care’**, ***f*_**ret**_ = 1, listed in Table 3A at SCCS (2021), for the skin care product type.

**Table 3:** Dermal assessment exposure by CRO1 and CR1 peloids

Parameter	Symbol	Value/formula	Unit
Skin area in contact with product	$A_{{{\text{skin}}}}$	15,670 (SCCS, 2021)	cm^2^
Amount of product in contact with skin	$Q_{{{\text{prod}}}}$	416 (SCCS, 2021)	g
Concentration of the substance in product (peloid)	*C*	–	weight %
Body weight	bw	60 (SCCS, 2021)	Kg_bw_
Number/frequency of applications	*n*	1/d	/d
Dermal absorption by perspiration	*D*_ap_	–	weight %
Dermal load	$L_{{{\text{der}}}}$	$L_{{{\text{der}}}} \, = \,\left( {Q_{{{\text{prod}}}} \, \times \, Fc_{{{\text{prod}}}} } \right)/A_{{{\text{skin}}}} \, \times \,1000$	mg/cm^2^
Dermal dose	$D_{{{\text{der}}}}$	$D_{{{\text{der}}}} \, = \,\left( {Q_{{{\text{prod}}}} \times \, Fc_{{{\text{prod}}}} \, \times \, n} \right)/bw\, \times \,1000$	mg/kg_bw_/d
Systemic exposure dosage	SED	$E_{{{\text{product}}}} \, \times \, C/100\, \times \,D_{{{\text{Ap}}}} /100$	mg/kg_bw_/d
No observed adverse effect level	NOAEL	Ni = 5 (Ma'or et al., 2015) and Cr = 2.5 (Ma'or et al., 2015)	mg/kg_bw_/d
Risk assessment	MoS	NOAEL/SED	If ≥ 100 substance is safe

The external dermal exposure (*E*_dermal_) per day for a product category $x$ can be calculated according to:

1 $$E_{{{\text{dermal}} x}} = C_{x} \times q_{x} \times f_{{{\text{ret}} x}} ,$$

${E}_{\mathrm{dermal} x}$ (mg/day): external exposure available for dermal uptake from product category$x$; $x$: product category; ${C}_{x}$ (mg/g): concentration/fraction of a substance in a product category$x$; ${q}_{x} (\mathrm{g}/\mathrm{day})$: amount of product category $x$ that is applied/received per day; ${f}_{\mathrm{ret} x}$: retention factor specific to product category $x$

Since the daily amount and retention factor are specific to the product category, when multiplied, they yield the daily exposure effective amount for it (*E*_product_ = *q*_*x*_ × *f*_ret*x*_).

The calculation of the Systemic Exposure Dose (SED, mg/Kg_bw_/d) was based in the SCCS suggested possibility by the percentage dermally absorbed, depending on the amount of finished product applied on the skin.

The calculation of the Systemic Exposure Dose (SED) for first-tier exposure via skin is based on three parameters: the estimated daily exposure to a cosmetic product (*E*_product_, mg/kg_bw_/day); C (%) concentration of the substance under study in the finished cosmetic product on the application site; and the assumed dermal absorption that is expected to occur in real-life conditions (*D*_ap_, %). Equation 2. is utilized to calculate the SED, expressed in milligrams of the substance per kilogram of body weight per day (mg/Kg_bw_/d).

Calculation formula:

2 $${\text{SED}} = E_{{{\text{product}}}} \times \frac{C}{100} \times \frac{{D_{{{\text{Ap}}}} }}{100},$$

The risk characterization was conducted by calculating the safety margin, using Eq. 3. The MoS is the ratio between the dose descriptor for the systemic exposure to a substance (usually historical NOAEL) and an estimate of the exposure (SCCS, 2021). Values greater than or equal to 100 indicate that substances can be considered safe and can be applied to children as well.

Calculation formula:

3 $$\mathrm{MoS} = \frac{\mathrm{NOAEL}}{\mathrm{SED}},$$

The NOAEL, or Non-Observed Adverse Effect Level, is the level of exposure at which no biologically significant increase in the frequency or severity of any adverse effects is observed using the tested method employed in the experiment. Several reports presenting NOAEL values for hazardous substances are available. They are usually obtained from repeated oral dosage toxicity studies conducted in experimental animals.

The “worst case scenario” calculation is recommended for substances without NOAEL values, using the known NOAEL values despite the lack of precise absorption data for the intended use of the studied substance. The following values were considered: for nickel 5 mg/Kg_bw_/day, measured for NiSO_4_ in a 2-year oral rat study; and for chromium 2.5 mg/kg_bw_/day, without distinguishing the different types of valences, based on intestinal absorption studies in both humans and animals (Höglund et al., 2012; Ma'or et al., 2015).

Since peloids are applied directly to the skin, this perspiration test can be used as a first-tier worst case model to evaluate exposure to trace elements and cations. The exposure will be expressed by the dermal load (*L*_der,_ mg/cm^2^) (Eq. 4) caused by perspiration and dermal dose (*D*_der,_ mg/Kg/d) (Eq. 5), which can determine the extent of contact with trace elements. For calculation of the dermal dose requires consideration of the frequency of application, dosage of the product ingredient and skin contact area.

Calculation formulas:

4 $$L_{{{\text{der}}}} = \frac{{Q_{{{\text{prod}}}} \times Fc_{{{\text{prod}}}} }}{{A_{{{\text{skin}}}} }} \times 1000,$$

5 $$D_{{{\text{der}}}} = \frac{{Q_{{{\text{prod}}}} \times Fc_{{{\text{prod}}}} \times n}}{{{\text{bw}}}} \times 1000,$$

The data for dermal assessment exposure evaluation are compiled in Table 3.

## Results

### Mineralogical characterization of the peloids

The mineralogical composition of CR1 and CRO1 peloids is shown in Table 4. Upon comparison of the raw clay (BV3) with the maturated ones, both CR1 and CRO1 samples are found to be enriched with phyllosilicates (more than 80%), being close to monomineralic samples. From the raw to the manipulated/treated material, the content of calcite does not change, iron oxides/hydroxides decrease, and feldspars become undetected.

**Table 4:** Mineralogical composition (wt.%) of Benavila Bentonite, CRO1 and CR1 peloids (the percentages of clay minerals are of the fraction smaller than 2 µm)

Mineral	BV3 (Rebelo et al., 2011)	CRO1	CR1
Quartz	2	2	1.5
K-Feldspar	1		
Anorthite	7		
Calcite	12	11	9
Dolomite	4		
Siderite	1		
Fe oxide/hydroxide	5	3	2.5
Hornblende	3		
Cristobalite	1		
Pyrite		2	
Phyllosilicates	64	82	87
Smectite	40	72	75
Illite–Smectite	5		
Illite	3		
Kaolinite	16	10	8
Scarbroite			4

**Fig. 1:**
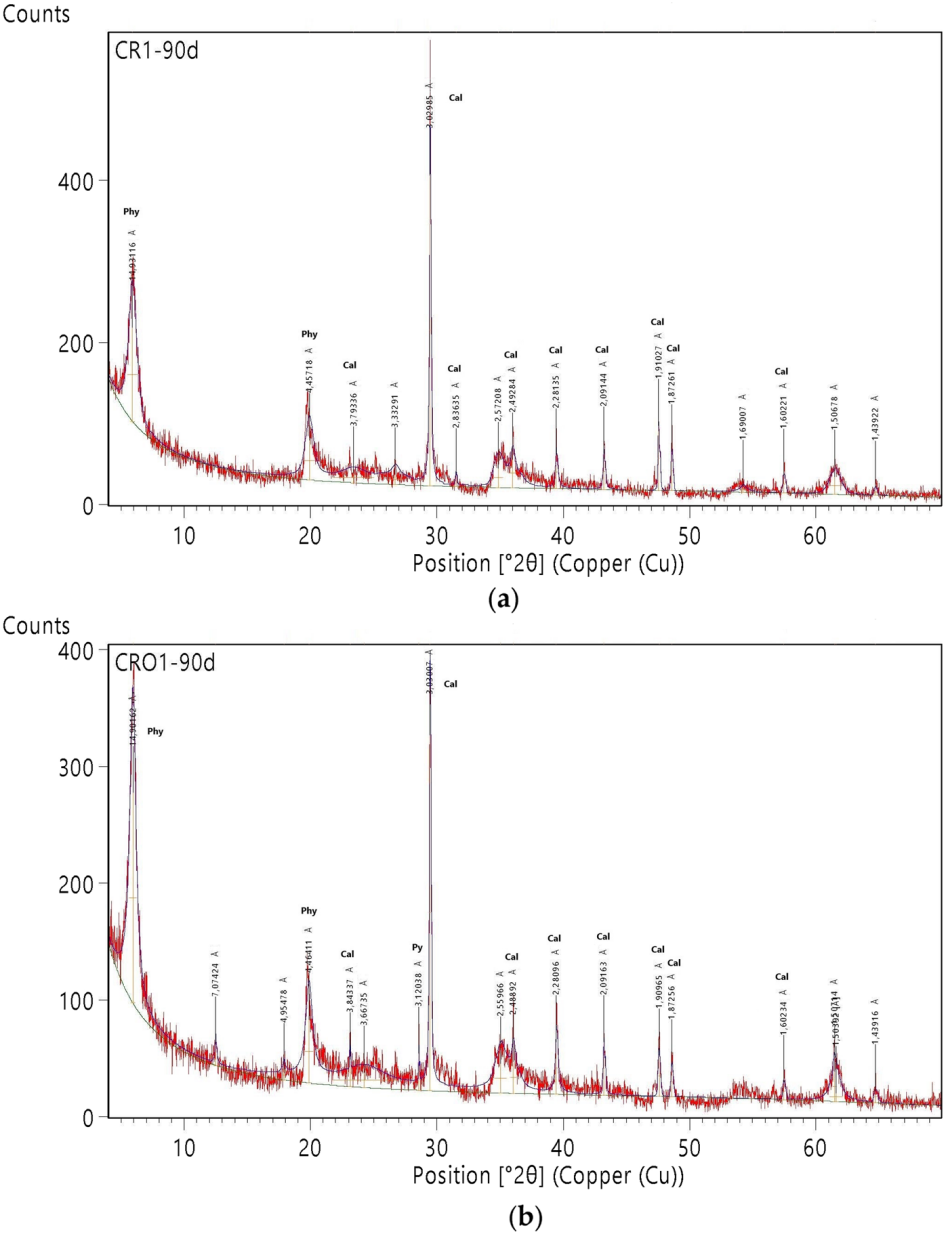
XRD diagrams, powder disoriented mounts. **a** CRO1 peloid; **b** CR1 peloid. (Phy: phyllosilicates; Cal: calcite; Py: pyrite)

**Fig. 2:**
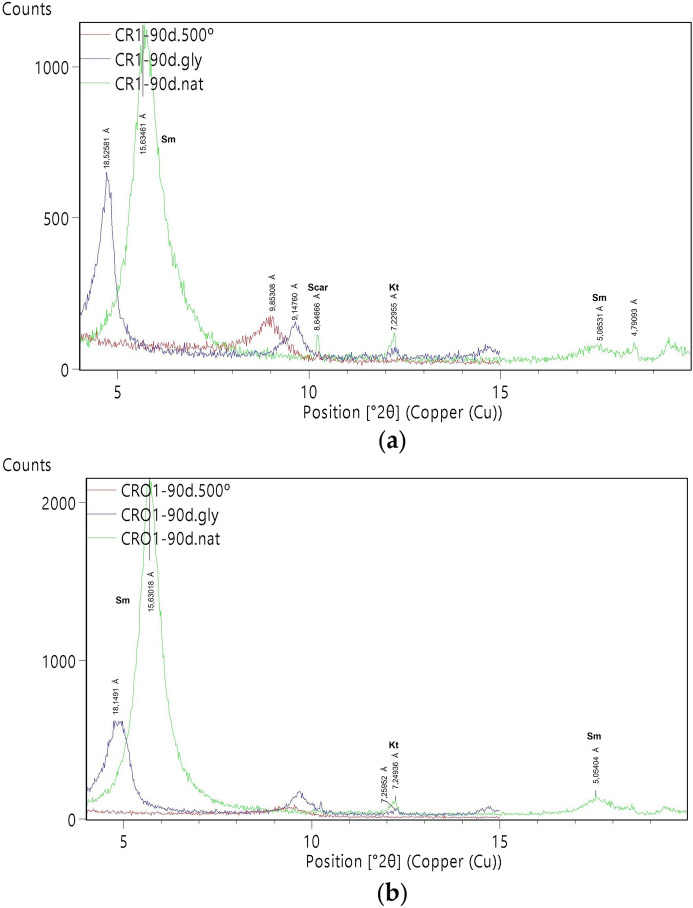
X-ray diffraction patterns of air-dried oriented aggregates of clay fractions. **a** CR1 peloid; **b** CRO1 peloid (Sm: smectite; Kt: kaolinite; Scar: scarbroite)

The smectites content in peloids CR1 and CRO1 is almost twice that of BV3, and their structural order is significantly higher in CRO1. Smectites are mainly of dioctahedral type, with d(060) values ranging between 1.493 and 1.502 Å. Illite–smectite was detected in the BV3 sample through an 11 Å diffraction band on natural oriented aggregate, which expanded to 13 Å on glycolated mount and collapsed to 10 Å when heated. Kaolinite was also present in the peloids, albeit in lesser amounts than in BV3, while illite (and illite–smectite) was absent (Figs. 1 and 2).

**Fig. 3:**
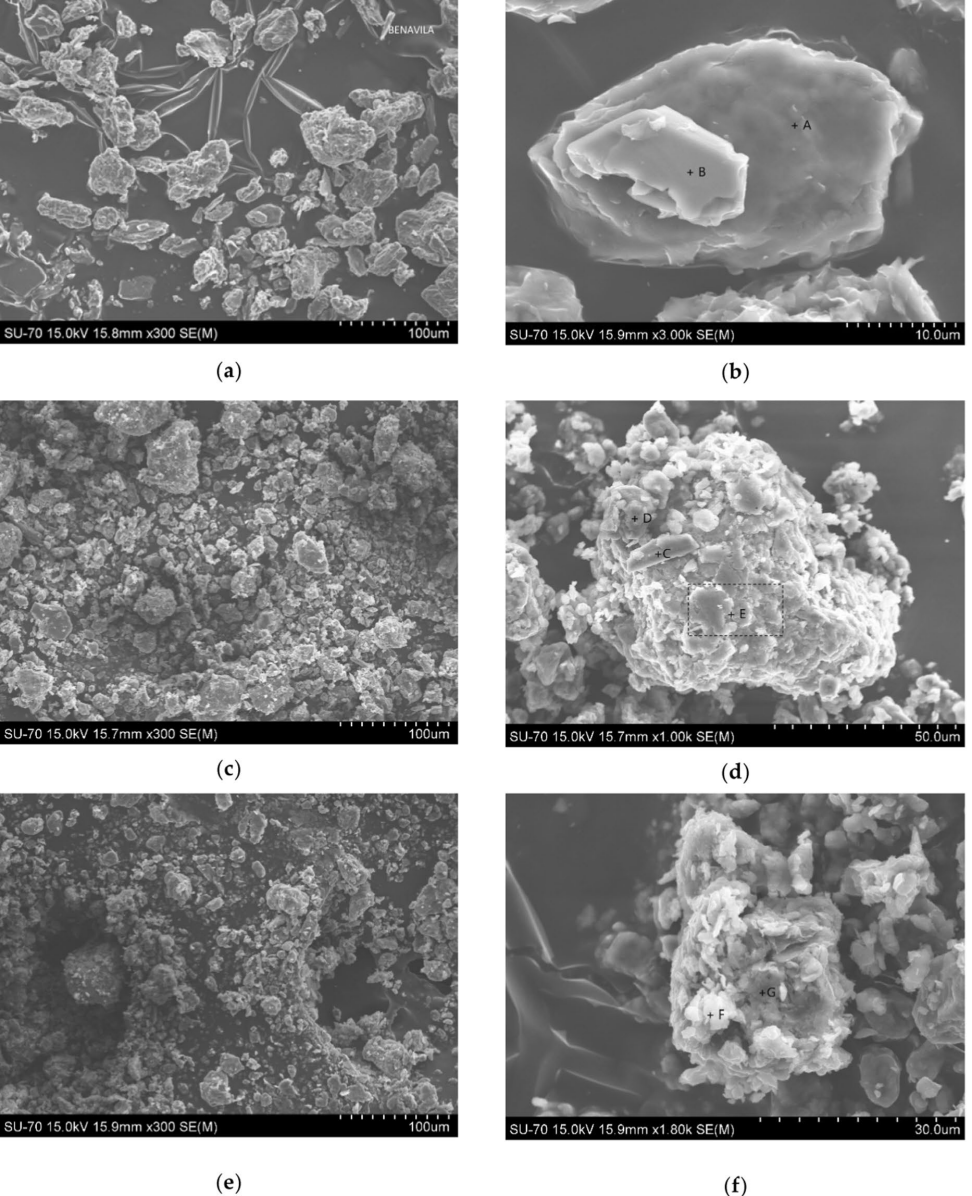
Comparative SEM images of Benavila bentonite (BV3), CRO1 peloid and CR1 peloid. **a** General view of aggregates in Benavila bentonite sample; **b** image of Benavila particles; **c** general view of aggregates in CR1 peloid; **d** image of CR1 peloid particles (smectite); **e** general view of aggregates in CRO1 peloid; **f** image of CRO1 peloid particles (smectite)

SEM images of Benavila clay particles and the two maturated peloids, CRO and CR, showed different morphologies (Fig. 3) as well as grain size distribution; maturated particles appear to be smaller showing more irregular shape. EDS analysis showed an abundance of Ca and Mg in all peloid samples and the presence of Na and K in smaller amounts (Table 5).

**Table 5:** SEM–EDS analysis (wt.%) results of Benavila bentonite (BV3), CRO1 and CR1 peloids

Sample	Point	Si	Al	O	Mg	Fe	Ca	Na	K	Cu	Cl
BV3	A	24.11	3.47	42.98	10.22	8.83	9.44	0.60	0.36	–	–
	B	27.84	7.00	46.67	3.55	13.77	0.68	0.25	–	0.23	–
CR1	C	24.66	2.47	46.48	10.06	7.69	8.64	–	–	–	–
	D	31.67	7.16	23.83	2.58	27.39	7.30	0.08	–	–	–
	E	26.90	7.53	44.19	3.64	14.14	3.07	0.34	–	–	0.20
CRO1	F	10.23	3.17	51.92	2.04	4.82	27.82	–	–	–	–
	G	23.01	7.33	54.99	3.71	7.07	3.89	–	–	–	–

These results allowed the smectites’ structural (crystallochemical) formula to be computed for each sample (Table 6).

**Table 6:** Structural formulas of smectite particles

Sample	Point	Formula
BV3	B	(Si_3.35_Al_0.65_) (Al_0.19_Fe_1.66_Mg_0.12_Cu_0.03_) (Mg_0.31_Na_0.03_Ca_0.08_) O_10_(OH)_2_
CR1	E	(Si_3.17_Al_0.83_) (Al_0.06_Fe_1.67_Mg_0.27_) (Mg_0.16_Na_0.04_Ca_0.36_) O_10_(OH)_2_
CRO1	F	(Si_3.37_Al_0.63_) (Al_0.44_Fe_1.03_Mg_0.53_) (Mg_0.01_Ca_0.57_) O_10_(OH)_2_

These structural formulas point to iron-rich smectites of a nontronite type, as determined by SEM/EDS. In maturated samples, Ca is the main exchangeable cation. Table 6 displays the original bentonite that was used as the raw material for preparing the peloids, as well as the two peloids that were maturated with different mineral-medicinal waters, inducing in different crystallochemical transformations, which explains the observed differences between them.

### Chemical characterization of the peloids

#### Bentonite interaction with mineral-medicinal waters

The supernatant collected after 90 days of maturation (Table 7) showed that some trace elements were below the detection limit for both peloids, such as Ag (0.2 µg/L), Be (0.5 µg/L), Bi (5 µg/L), Cd (0.4 µg/L), P(15.00 µg/L); Pb (0.5 µg/L), Sb (0.5 µg/L), Sn (0.5 µg/L), Th (3 µg/L), Tl (0.1 µg/L), U (0.1 µg/L) and Zn (5.0 µg/L).

**Table 7:** Supernatant chemical composition collected at 90 days (*d*) of maturation, analyzed by ICP-MS

Elements	Unit	d.l.^a^	Supernatant		Mineral-medicinal water	
			CRO1 (90d)	CR1 (90d)	Cró^b^ (Cirne, 2019)	Caldas da Rainha (Rebelo et al., 2015)
Ag	µg/L	0.2	< *d.l*	< *d.l*	0.53	–
Al	µg/L	5	47.0	< 0.5	6.4	13
As	µg/L	1	2.86	1.13	12.8	6
B	µg/L	10	163	50.6	–	< *d.l*
Ba	µg/L	0.5	134	66.4	< 1.3	38
Be	µg/L	0.5	< *d.l*	< *d.l*	0.60	–
Bi	µg/L	5	< *d.l*	< *d.l*	–	–
Ca	mg/L	0.2	36.3	369	3.9	305
Cd	µg/L	0.4	< *d.l*	< *d.l*	0.22	–
Co	µg/L	0.2	0.74	2.79	< 0.36	–
Cr	µg/L	0.2	6.14	5.98	< 2.1	21
Cu	µg/L	0.5	1.45	1.70	< 1.1	4
Fe	µg/L	5	52.2	8.95	26	5
K	mg/L	1	3.38	7.80	2.8	5
Li	µg/L	1	344	37.2	675	67
Mg	mg/L	0.2	65.3	484	0.2	61
Mo	µg/L	0.4	3.44	0.85	1.1	0.05
Mn	µg/L	0.5	65.8	426	21.1	19
Na	mg/L	0.2	99.8	928	98.7	662
Ni	µg/L	0.4	0.87	3.39	< 17.2	–
P	µg/L	15	< *d.l*	< *d.l*	–	–
Pb	µg/L	0.5	< *d.l*	< *d.l*	< 0.65	–
Sb	µg/L	0.5	< *d.l*	< *d.l*	< 0.14	–
Sn	µg/L	0.5	< *d.l*	< *d.l*	< 0.47	–
Sr	µg/L	0.5	368	4567	–	4618
Th	µg/L	3	< *d.l*	< *d.l*	–	–
Tl	µg/L	0.1	< *d.l*	< *d.l*	< 0.21	–
U	µg/L	0.1	< *d.l*	< *d.l*	–	0.19
V	µg/L	0.1	45.69	23.41	< 0.49	8.7
W	µg/L	0.5	24.99	2.46	76.9	2.0
Zn	µg/L	5	< *d.l*	< *d.l*	< 6.2	13.7

Chemical analysis references for the mineral-medicinal waters

^a^*d.l*.-detection limit

^b^LAIST/ISQ Analysis Report No 097/H/2011, 2011.07.05 and LAIST/ISQ Analysis Report No 02669–16, 2016.01.28, mean values

The CRO supernatant had higher content of Al, As, B, Ba, Fe, Li, Mo and V when compared with that of CR supernatant, whereas CR showed higher values of Ca, Co, Mg, Mn, Na and Sr. The concentration of chromium was similar in both peloids.

When compared with the mineral-medicinal water, the supernatant of the two maturation tanks showed several changes in trace elements concentration. The supernatant from maturation tank CRO1 mostly showed higher values in trace elements, except for Ag, Be, Cd and Pb. In the supernatant of maturation tank CR1, there was a marked enrichment in the elements B, Ba, Mg, Mn, Ni and Na.

The concentration of major elements, reported as oxides (wt %) SiO_2_, Al_2_O_3_, MnO and Na_2_O, increased in the solid phase of the peloids after 90 days of maturation, as shown in Table 8.The CR1 peloid showed significant increase in Na_2_O concentration (0.414%) and SO_3_ concentration (0.154%). Both peloids had an increase in loss on ignition, with similar values (CR1: 15.38% and CRO1: 15.36%). The concentrations of Fe_2_O_3_, MgO and CaO decreased after 90 days of maturation.

**Table 8:** Major element (wt.%) content in bentonite (BV3) and in the maturated peloids, by XRF

Major Elements	BV3	CRO1	CR1
SiO_2_	35.10	41.73	42.03
Al_2_O_3_	8.87	10.90	11.79
Fe_2_O_3_	14.21	13.34	12.47
CaO	19.39	11.34	10.84
TiO_2_	0.785	0.739	0.705
MnO	0.069	0.075	0.078
K_2_O	0.467	0.406	0.400
P_2_O_5_	0.025	0.025	0.023
MgO	6.33	5.49	5.21
Na_2_O	0.071	0.133	0.414
SO_3_	0.007	0.010	0.154
LOI^a^	14.39	15.36	15.38

^a^*LOI *loss on ignition

The trace elements (Table 9) also exhibited some changes. Chlorine, which was supplied by the mineral water (Table 2), was highly evident in the CR1 peloid (Cl: 2610 ppm).

**Table 9:** Trace elements (ppm) present in bentonite (BV3) and in the maturated peloids, by XRF

Minor Elements	BV3	CRO1	CR1
Ag	< *d.l*	< *d.l*	< *d.l*
As	< *d.l*	< *d.l*	< *d.l*
Ba	120	170	210
Bi	< *d.l*	< *d.l*	< *d.l*
Br	1.1	2.3	3.2
Cd	< *d.l*	< *d.l*	< *d.l*
Cl	< *d.l*	170	2610
Co	21.2	18.3	17.2
Cr	2140	1850	1760
Cu	93.7	44.4	46.9
Ga	9.0	11.3	11.0
Ge	< *d.l*	< *d.l*	< *d.l*
Mo	1.2	1.1	1.3
Ni	250.0	75.3	69.4
Nb	2.9	3.1	3.4
Pb	2.7	6.3	9.5
Rb	24.7	19.1	18.4
Sb	< *d.l*	< *d.l*	< *d.l*
Sc	43.0	47.5	51.4
Se	< *d.l*	< *d.l*	< *d.l*
Sn	4.3	6.9	6.3
Sr	67.8	83.3	99.2
Te	< *d.l*	< *d.l*	< *d.l*
Th	< *d.l*	< *d.l*	< *d.l*
Y	11.0	11.7	11.3
U	1.4	< *d.l*	1.8
V	120	110	110
Zn	62.0	47.1	45.3
Zr	46.7	45.5	46.8

< *d.l. *detection limit

After 90 days of the maturation process of BV3, elements such Cr, Co, Cu, Ni, Rb, Sn, V and Zn decreased, while Ba, Br, Ga, Nb, Pb, Sc and Sr increased.

The usual values reported in the literature for CEC of bentonite, vary significantly, from 20 to 130 meq/100 g [Rebelo et al., 2011 and others therein]. In this study, the CEC of the raw material is tendentially low (45 meq/100 g), although it slightly increases with maturation. As maturation progresses, exchangeable Ca and Mg decrease, while K and Na increase, suggesting that the latter are competing for the interlayer spaces (Table 10).

**Table 10:** Exchangeable cations and cation exchange capacity (CEC) values (meq/100 g)

	BV3	CRO1	CR1
Ca^2+^	685	370	303
Mg^2+^	163	5	5
Na^+^	3	143	150
K^+^	6	13	61
$\sum \mathrm{cation}$	857	531	519
CEC	45	66	57

#### Peloids interaction with artificial sweat

The chemical composition of the artificial perspiration solution is presented in Table 11. The solution is composed mainly of Ag, Al, Ba, Ca, Cr, Fe, K, Li, Mg, Mn, Na, Ni, P, Pb, Sn, Sr, V and Zn. It is noteworthy that the solution contains detectable concentrations of potentially toxic elements such as Cr, Pb and Zn.

**Table 11:** Chemical composition (µg/L, mg/L) of the artificial perspiration solution (APS) by ICP-MS

	Unit	d.l	Artificial perspiration
Ag	µg/L	0.30	1.04
Al	µg/L	13.5	25.04
As	µg/L	1.00	< *d.l*
B	µg/L	5.00	< *d.l*
Ba	µg/L	1.00	5.47
Be	µg/L	2.00	< *d.l*
Bi	µg/L	5.00	< *d.l*
Ca	mg/L	0.30	0.45
Cd	µg/L	0.15	< *d.l*
Co	µg/L	0.50	< *d.l*
Cr	µg/L	1.00	1.82
Cu	µg/L	1.00	< *d.l*
Fe	µg/L	10.0	21.21
K	mg/L	1.00	2.73
Li	µg/L	2.00	5.32
Mg	mg/L	0.10	19.43
Mo	µg/L	0.30	< *d.l*
Mn	µg/L	2.00	5.08
Na	mg/L	0.10	> 2000
Ni	µg/L	0.50	0.57
P	µg/L	60.0	157.4
Pb	µg/L	0.70	1.93
Sb	µg/L	0.20	< *d.l*
Se	µg/L	3.00	< *d.l*
Sn	µg/L	0.40	9.96
Sr	µg/L	1.00	4.95
Tl	µg/L	0.50	< *d.l*
Th	µg/L	1.00	< *d.l*
V	µg/L	1.00	1.24
W	µg/L	0.50	< *d.l*
Zn	µg/L	4.00	6.39

*d.l. *detection limit

The concentration of soluble elements in bentonite (BV3), CRO1 and CR1 peloids using the artificial perspiration solution is shown in Table 12. The results indicate that As, B, Co, Cu and W were the most relevant soluble chemical elements in the clay samples, while lithium was found to be dissolved at a higher concentration in the CRO1 peloid, and manganese was dissolved at a higher concentration in the CR1 peloid.

**Table 12:** Concentration (µg/L, mg/L) of the soluble elements of the bentonite (BV3), CRO1 and CR1 peloids, using artificial perspiration solution (APS), by ICP-MS

	d.l	Unit	BV3				CRO1				CR1			
			S1	S2	S3	Mean ± SE, *n* = 3	S1	S2	S3	Mean ± SE, *n* = 3	S1	S2	S3	Mean ± SE, *n* = 3
Ag	0.30	µg/L	1.06	1.04	1.05	1.05 ± 0.01	1.42	1.13	1.12	1.22 ± 0.10	1.13	1.08	1.08	1.10 ± 0.02
Al	13.5	µg/L	494.64	409.46	536.69	480.26 ± 37.42	2302.40	1893.43	648.04	1614.62. ± 497.50	977.58	589.69	742.27	769.84 ± 112.82
As	1.00	µg/L	1.53	1.46	1.57	1.52 ± 0.03	1.81	1.35	1.37	1.51 ± 0.15	1.29	1.30	1.31	1.30 ± 0.01
B	5.00	µg/L	9.04	5.87	8.19	7.70 ± 0.95	10.15	10.40	10.07	10.21 ± 0.10	6.52	10.62	12.51	9.89 ± 1.77
Ba	1.00	µg/L	207.78	205.01	217.88	210.22 ± 3.91	275.78	245.06	246.06	255.63 ± 10.08	321.06	290.14	306.76	305.99 ± 8.94
Be	2.00	µg/L	< *d.l*	< *d.l*	< *d.l*	–	< *d.l*	< *d.l*	< *d.l*	–	< *d.l*	< *d.l*	< *d.l*	–
Bi	5.00	µg/L	< *d.l*	< *d.l*	< *d.l*	–	< *d.l*	< *d.l*	< *d.l*	–	< *d.l*	< *d.l*	< *d.l*	–
Ca	0.30	mg/L	101.44	99.53	106.45	102.47 ± 2.06	94.24	89.06	91.38	91.56 ± 1.50	111.42	114.43	122.04	115.96 ± 3.16
Cd	0.15	µg/L	< *d.l*	< *d.l*	< *d.l*	–	< *d.l*	< *d.l*	< *d.l*	–	0.18	0.18	0.23	0.20 ± 0.02
Co	0.50	µg/L	0.76	0.55	0.66	0.66 ± 0.06	0.88	0.74	0.56	0.73 ± 0.09	1.25	1.13	1.34	1.24 ± 0.06
Cr	1.00	µg/L	12.78	10.77	11.46	11.67 ± 0.59	42.06	34.06	21.20	32.44 ± 6.08	14.40	16.83	12.78	17.58 ± 2.09
Cu	1.00	µg/L	8.31	8.96	9.19	8.82 ± 0.27	10.80	14.27	12.22	12.43 ± 1.01	10.50	9.86	10.18	10.18 ± 0.19
Fe	10.0	µg/L	1072.84	895.67	883.18	950.56 ± 61.24	2862.80	2622.97	1044.24	2176.67 ± 570.43	1473.45	913.06	1136.92	1174.48 ± 162.86
K	1.00	mg/L	4.83	4.93	5.28	5.01 ± 0.14	4.25	3.96	3.99	4.07 ± 0.09	4.25	4.32	4.82	4.46 ± 0.18
Li	2.00	µg/L	7.28	6.29	6.86	6.81 ± 0.29	101.82	89.20	88.61	93.21 ± 4.31	13.12	14.05	13.99	13.69 ± 0.29
Mg	0.10	mg/L	150.33	148.73	160.86	153.31 ± 3.81	149.68	131.36	133.96	138.33 ± 5.72	148.86	152.88	164.57	155.44 ± 4.71
Mo	0.30	µg/L	0.43	0.36	0.33	0.37 ± 0.03	0.61	0.41	0.58	0.53 ± 0.06	0.39	0.35	0.52	0.42 ± 0.05
Mn	2.00	µg/L	44.42	45.83	49.60	46.62 ± 1.55	55.87	41.28	34.17	43.77 ± 6.39	240.45	238.88	280.76	253.37 ± 13.71
Na	0.10	mg/L	> 2000	> 2000	> 2000	–	> 2000	> 2000	> 2000	–	> 2000	> 2000	> 2000	–
Ni	0.50	µg/L	2.77	1.58	1.88	2.08 ± 0.36	3.33	4.45	4.43	4.07 ± 0.37	2.48	3.47	2.59	2.85 ± 0.31
P	60.0	µg/L	39.80	47.08	46.78	44.55 ± 2.38	71.59	24.91	24.86	40.46 ± 15.57	53.73	45.82	57.90	52.48 ± 3.54
Pb	0.70	µg/L	0.73	0.79	0.72	0.75 ± 0.02	0.98	1.07	0.93	0.99 ± 0.04	0.93	0.82	0.79	0.84 ± 0.04
Sb	0.20	µg/L	< *d.l*	< *d.l*	< *d.l*	–	< *d.l*	< *d.l*	< *d.l*	–	< *d.l*	< *d.l*	< *d.l*	–
Se	3.00	µg/L	< *d.l*	< *d.l*	< *d.l*	–	< *d.l*	< *d.l*	< *d.l*	–	< *d.l*	< *d.l*	< *d.l*	–
Sn	0.40	µg/L	0.63	1.27	0.88	0.93 ± 0.19	2.28	0.79	0.92	1.33 ± 0.48	1.00	1.33	0.78	1.04 ± 0.16
Sr	1.00	µg/L	848.01	834.88	890.06	857.65 ± 16.64	834.85	736.28	754.59	775.24 ± 30.27	1102.77	1138.79	1218.53	1153.36 ± 34.20
Tl	0.50	µg/L	< *d.l*	< *d.l*	< *d.l*	–	< *d.l*	< *d.l*	< *d.l*	–	< *d.l*	< *d.l*	< *d.l*	–
Th	1.00	µg/L	< *d.l*	< *d.l*	< *d.l*	–	< *d.l*	< *d.l*	< *d.l*	–	< *d.l*	< *d.l*	< *d.l*	–
V	1.00	µg/L	20.37	19.07	27.28	22.24 ± 2.55	24.73	22.99	22.57	23.43 ± 0.66	20.04	20.18	28.55	22.92 ± 2.81
W	0.50	µg/L	2.56	2.21	2.55	2.44 ± 0.11	1.72	1.44	1.45	1.53 ± 0.09	1.38	1.44	1.45	1.42 ± 0.02
Zn	4.00	µg/L	< *d.l*	9.24	< *d.l*	–	4.11	9.00	5.40	6.17 ± 1.46	4.44	4.54	< *d.l*	–

*d.l.-*detection limit

The elements Mo, Pb, P and Sn are present in high concentration in the artificial perspiration solution (APS), and their solubility cannot be evaluated (Table 11). It is noteworthy that Ag is present in the APS at similar concentrations to those in the analyzed samples, suggesting a low solubility of this element in the solution.

It was not possible to evaluate the solubility of Zn in BV3 and CR1 because some data from the replicate samples were below the detection limit for zinc. However, with data from the replicate samples for the CRO1 peloid, it can be assumed that there were exchanges between the solution and the peloid (Table 12).

The trace elements Be, Bi, Sb, Se, Tl and Th are below the detection limit in all clay samples and in the sweat solution. Only Cd was soluble in the CR1 peloid.

The exchangeable cations Ca^2+^, Mg^2+^, K^+^, and Na^+^ are present in both perspiration solution and the clayey samples, allowing for easy ionic exchanges between the solid and liquid phases. The perspiration solution is sodic (> 2000 mg/L) and the leached clayey samples present similar concentrations of this element. While calcium and magnesium are soluble in sweat in all clayey samples, potassium presents a lower solubility in the artificial fluid.

### Exposure assessment of chromium and nickel

The dermal exposure assessment of these peloids and the dermal uptake of Cr and Ni by the dermal route are presented in Table 3.

The calculation of the daily exposure amount per body weight was based on the concentration of Ni and Cr present in the peloids, expressed in mass % (Table 9).

Following the SCCS (2021) recommendations for dermal exposure calculation, the daily exposure for Cr was found to be 769.60 mg/day, 732.16 mg/day and 890.24 mg/day for CRO1, CR1 and BV3, respectively (calculated using Eq. 1). For Ni, the calculated values were 31.32 mg/day (CRO1), 28.87 mg/day (CR1) and 104.00 mg/day (BV3). The external dermal exposure of these trace elements was lower in the peloids (Table 13).

**Table 13:** Peloids daily exposure (mg/day)

	*C* (mg/g)	*q* (g/day)	*f*_ret_	*E*_dermal_ (mg/day)
*CRO1*				
Chromium	1.85	416	1	769.60
Nickel	0.0753	416	1	31.32
*CR1*				
Chromium	1.76	416	1	732.16
Nickel	0.0694	416	1	28.87
*BV3*				
Chromium	2.14	416	1	890.24
Nickel	0.25	416	1	104.00

The resulting concentration of the elements from BV3, CRO and CR peloids, using artificial perspiration, indicates a low systemic exposure dose, which is below the non-observed adverse effect level of 5 mg/Kg_bw_/day (Ni) and 2.5 mg/kg_bw_/day (Cr).

The toxicological profile of these peloids for the dermal route, particularly for prohibited elements such as Cr and Ni, is classified as having negligible absorption or not being relevant, since the margin of safety (MoS) is above 100 (Table 14).

**Table 14:** Exposure assessment and safety evaluation of chromium and nickel content

				BV3	CRO1	CR1
Concentration of concerned substance in peloid (Table 9)	*C*	Weight %	Nickel	0.0250	0.0075	0.0069
			Chromium	0.214	0.185	0.176
Dermal absorption by perspiration (Table 12)	*D*_Ap_	Weight %	Nickel	2.08 × 10^−6^	4.07 × 10^−6^	2.85 × 10^−6^
			Chromium	1.17 × 10^−5^	3.24 × 10^−5^	1.76 × 10^−5^
Dermal load (Eq. 4)	*L*_der_	mg/cm^2^	Nickel	5.52 × 10^−5^	1.08 × 10^−4^	7.57 × 10^−5^
			Chromium	3.10 × 10^−4^	8.61 × 10^−4^	4.67 × 10^−4^
Dermal dose (Eq. 5)	*D*_der_	mg/Kg_bw_/d	Nickel	0.014	0.028	0.020
			Chromium	0.081	0.225	0.122
Systemic Exposure Dosage (Eq. 2)	SED	mg/Kg_bw_/d	Nickel	7.28 × 10^−14^	8.58 × 10^−14^	3.96 × 10^−14^
			Chromium	2.02 × 10^−11^	1.35 × 10^−10^	3.77 × 10^−11^
Risk assessment (Eq. 3)	MoS	If ≥ 100 substance is safe	Nickel	3.43 × 10^13^	2.91 × 10^13^	6.32 × 10^13^
			Chromium	2.47 × 10^11^	3.70 × 10^10^	1.32 × 10^11^

## Discussion

The maturation of Benavila bentonite using different mineral-medicinal waters for 90 days led to noticeable chemical and mineralogical changes, as evidenced by the resulting peloids. Such rapid transformations have been documented by several authors, including Tateo et al. (2010), who observed significant changes in all the samples during the first month of maturation and the subsequent two months (Concerning the clay mineral content, kaolinite decrease is a consequence of interaction with these highly mineralized waters, with a high content of alkalis, favoring smectitization (Table 4) (Quintela et al., 2012). The presence of pyrite may result from the interaction with the sulfurous water (Quintela et al., 2012; Schlosser et al., 2016).

A few morphological changes were identified, specifically a decrease in particle size and more irregularly shaped particles (SEM, Fig. 2), which may be related with the used sulfurous waters (chloride and sodium rich; bicarbonate and sodium rich), the stirring process that fragmentizes the minerals to smaller particles and the presence of cations able to occupy the interlayer spaces of the smectite (Carbajo & Maraver, 2018).

These structural formulas point to an iron-rich smectite of a nontronite type. In the raw clay, Mg is the main exchangeable cation, but in the maturated samples, Ca replaces Mg in this position. The concentration of Mg^2+^ decreases, while Ca^2+^ becomes the main exchangeable cation. Na^+^ and K^+^ also begin to compete for the interlayer spaces, which is influenced by the hydration energy of the interlayer cation and changes in the electrostatic surface potentials (Table 10) (Brigatti et al., 2006).

Although the supernatant of the CR1 tank is richer in Ca (369.39 mg/L) when compared to the CRO1 supernatant (36.27 mg/L), this enrichment does not seem to result directly from the composition of the mineral-medicinal water. When we compare the BV3 Ca-content (102.47 mg/L) with CRO1 (91.56 mg/L) and CR1 (115.96 mg/L), BV3 appears to be the major Ca contributor (Table 7 and Table 10).

The decrease in Ca^2+^ and Mg^2+^ in CRO1 and CR1 may result from the partial dissolution of dolomite and calcite (Table 4) (Sánchez-Espezo et al., 2015). After 90 days of maturation, the amount of exchangeable Na^+^ and K^+^ increased, consistent with the mineralogical changes and supernatant chemical composition (Table 7). The increase in Na^+^ is related to the mineral-medicinal water composition (Rebelo et al., 2015), promoting the exchange of Ca^2+^ with Na^+^ in the smectite interlayer.

The sodium content in all three samples exceeds 2000 mg/L, which is easily dissolved not only due to the characteristics of the artificial perspiration solution (Table 11), but also because of the contribution of the maturation water (CR1(Na): 928.23 mg/L; CRO1 (Na): 99.82 mg/L) as well as its concentration in the maturated peloids (CR1 (Na_2_O): 0.414%; CRO1 (Na_2_O): 0.133%).

In the case of the lithium-rich CRO1 peloid, its high solubility in artificial sweat (concentration of 93.21 µg/L) demonstrates the significant effect of the mineral-medicinal water characteristics (as shown in Table 7) on the maturation process. Soluble Mn is more elevated in CR1, and the elements’ source seems to be the mineral water (CR1 supernatant 425.75 µg/L).

The solubility of elements in the artificial perspiration for BV3, CRO1 and CR1 differs due to the chemical composition of the mineral-medicinal water and the relative amount of smectite in the mineralogical composition (as shown in Table 15).

**Table 15:** Distribution of elements leached from BV3, CRO and CR peloids, using artificial perspiration

Smectite		Content of the leached elements
40%	BV3	Na > Fe > Sr > Al > Ba > Mg > Ca > Mn > V > Cr > Cu > B > W > K > As > Ni > Li > Co > Mo > Ag
72%	CRO1	Na > Fe > Al > Sr > Ba > Mg > Ca > Li > Mn > Cr > V > Cu > B > Ni > W > As > K > Co > Mo > Ag
75%	CR1	Na > Fe > Sr > Al > Ba > Mn > Mg > Ca > V > Cr > Cu > B > Li > Ni > K > W > As > Co > Mo > Cd > Ag

The influence of the mineral-medicinal water in the maturation process is relevant for the content of some leached elements, such as Li in CRO1 and Mn in the CR1 peloid.

Lithium has been used for decades to treat mental health disorders, although its specific function is not known, and there is low-quality evidence suggesting its benefits and human tolerability (World Health Organization, 2020). Some medical studies have revealed the favorable impact of drinking water rich in lithium on the treatment of dementia and depressive symptoms, but also the adverse impact on interpersonal violence (Kessing et al., 2017; Shimodera et al., 2018), and side effects such as thyroid function alteration (Broberg et al., 2011) or kidney problems (Gupta et al., 2019). Most health studies on the impact of lithium consider the source of lithium in the water intake [Neves et al., 2020 and authors therein].

The concentration of lithium, 93.21 ± 4.31 µg/L, is below the recommended therapeutic daily dosage intake (Neves et al., 2020; World Health Organization, 2020). The transdermal delivery of lithium from the CRO1 peloid does not pose a potential risk of toxicity for pelotherapy use, since the possibility of crossing the stratum corneum and reaching the bloodstream is negligible.

Manganese stands out in the CR1 peloid when compared to the CRO1 peloid, probably due to the interaction process of the BV3 bentonite with the water typology and the ability of smectite clays to adsorb metals (Khalil et al., 2018). The health impact of manganese, as an essential element, has also been surveilled, and it is known that the intake from drinking water is lower than the intake from food. Its beneficial effects are observed on cartilage and bone formation, antioxidative capacity and wound healing. However, some studies have documented adverse health effects when manganese levels are either in excess or deficient (Khalil et al., 2018; Mattison et al., 2017). The passive transdermal delivery of manganese from the CR1 peloid is also negligible. Only the use of an electric dermal administration technique (iontophoresis) on a cationic manganese solution has revealed an improvement in the passage of the Mn through the stratum corneum barrier (Ito et al., 2014).

The elements Fe (2176.67 and 1174.48 µg/L), Al (1614.62 and 769.84 µg/L), Sr (775.24 and 1153.36 µg/L) and Ba (255.63 and 305.99 µg/L) exhibited the highest concentrations in artificial sweat of the two peloids, CRO1 and CR1.

According to the Scientific Committee on Consumer Safety (SCCS) report on the safety of Aluminium (SCCS/1613/19), substances rich in Al that are used to prevent sweat from reaching the surface of the skin form chemically inert complexes with basic components of sweat and skin once they are applied to the skin. Moreover, the high molecular weight and high positive charges of these substances limit the potential for skin penetration through the stratum corneum. Some studies have measured the blood and urine concentration of 26Al in an applied topical dose, revealing low levels. The level of Al on clothes and equipment was also measured, providing evidence that the applied dose was lost from the surface of the skin. The SCCS (2021) considers 6.25% Al concentration in non-spray antiperspirants and 10.60% in spray antiperspirants as safe levels (SCCS, 2019).

Barium toxicity is produced by free cation and may cause adverse health effects due to its solubility (SCHER, 2012). However, there is scarce information in the literature about the harmful effects of Ba and its biological significance. Omata et al. (2018) present a direct correlation of Ba absorption with melanin, while Johnson et al. (2018) consider the safe use of substances with Ba only if formulated to be non-irritating (Johnson et al., 2018; Omata et al., 2018). CR1 and CRO1 abrasiveness is low (Quintela et al., 2014), so there is no risk of promoting skin irritation. However, the risk assessment for melanin alterations should be monitored with every use.

Some studies have identified Mg as the main element responsible for improving the psoriatic state and as having an anticarcinogenic effect on tissues (Matz et al., 2003). Sasaki et al. (2017) studied the wound-healing effect of Mg-smectite on tissue regeneration and considered it a potential candidate material for wound treatment due to the beneficial effects of released Mg^2+^ and Si^4+^ (Sasaki et al., 2017).

Calcium and Mg were present in higher concentrations in the CR1 peloid (115.96 ± 3.16 mg/L and 155.44 ± 4.71, respectively). The levels of trace elements prohibited in cosmetics, namely As, Cd, Cr, Ni, P, Pb, SB, Se, Te, and Tl, and their migration rates indicate that dermal exposure to CRO1 and CR1 peloids at 45 °C(± 2 °C) was within the precautionary safety limit established in Article 17 of EC Regulation 1223/2009 for the permitted conditions for the presence of traces of prohibited substances.

Chromium is a prohibited substance due to its carcinogenic action. Its safety depends on chromium speciation and it is recommended to be measured without differentiating Cr^3+^ (of natural origin) from the hexavalent form (Cr^6+^) of anthropogenic origin (Höglund et al., 2012). Other complementary studies should be done to evaluate chromium speciation. The EU standard identifies a safe limit for Cr of 1 ppm, and the content released from all samples was below this limit. The element Cd is also considered a prohibited substance in cosmetics, and it is released in the CR1 peloid, although it is not detectable in BV3 and supernatant.

The toxicological profile for nickel, presented by the Agency for Toxic Substances and Disease Registry (ATSDR, 2005), indicates that acute exposure to Ni results in skin irritations (dermatitis) and hypersensitivity. However, there are no studies regarding adverse reactions or other effects in humans after dermal exposure.

## Conclusions

The health risk associated with exposure to CRO1 and CR1 peloids, in terms of metals bioaccessibility, may have a negligible effect on risk estimation. This study has revealed that CR1 and CRO1 peloids can be safely used because they do not pose a possible source of human exposure to heavy metals. The amounts extracted from the studied samples were found to be extremely low and, in some cases, undetectable, assuming a safe linear dose–response relationship.

The elements soluble in the artificial perspiration for BV3, CRO1 and CR1 are different because of the chemical composition of the mineral-medicinal water and the relative amount of smectite in the mineralogical composition. The influence of the mineral-medicinal water in the maturation process is relevant for the content of some leached elements.

Calcium and Mg were in higher concentrations in the CR1 peloid. The levels of trace elements prohibited in cosmetics, namely As, Cd, Cr, Ni, P, Pb, SB, Se, Te and Tl, and their migration rates were within the precautionary safety limit, established in Article 17 of EC Regulation 1223/2009 for the permitted conditions for the presence of traces of prohibited substances.

The results from this bioaccessibility study mimetizing the phenomenon of the sudation effect and simultaneous vasodilation of the skin in pelotherapy showed the importance of the chemical composition of thermal water to the peloids’ chemical characteristics. Peloids are therapeutic agents that exert thermal and chemical effects on the skin through the stratum corneum. The dissolved ions in peloids may play a crucial role facilitating osmotic cellular processes when they come into contact with the skin. Relevant and reliable information about dermal exposure and the identification of some elements that may enter in the systemic circulation can be assessed. Therefore, this analytical procedure should be considered as a suitable quality control method for peloids and clayey raw materials.

## Supplementary Information

Below is the link to the electronic supplementary material.

Supplementary file1 (XLSX 15 KB)
